# Protein and Long-Chain n-3 Polyunsaturated Fatty Acids Recovered from Herring Brines upon Flocculation and Flotation—A Case Study

**DOI:** 10.1021/acssuschemeng.2c06795

**Published:** 2023-04-18

**Authors:** Bita Forghani, Mihaela Mihnea, Tore C. Svendsen, Ingrid Undeland

**Affiliations:** † Food and Nutrition Science, Biology and Biological Engineering, Chalmers University of Technology, SE-41296 Gothenburg, Sweden; ‡ Perception & Design Unit, Department of Material and Surface Design, RISE Research Institutes of Sweden, SE-41296 Gothenburg, Sweden; § Bio-Aqua A/S, Stroebjergvej 29, DK-3600 Frederikssund, Denmark; ∥ Aquarden Technologies ApS, Industrivej 17, 3320 Skævinge, Denmark

**Keywords:** marination brine, protein recovery, carrageenan, circular economy, wastewater

## Abstract

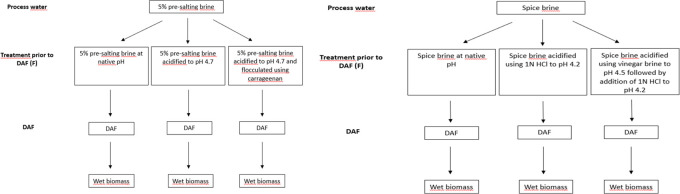

A novel integrated process for recovery of protein-enriched biomasses from 5% presalting brines and spice brines of herring (*Clupea harengus*) was investigated by combining carrageenan- and/or acid-driven flocculation (F) plus dissolved air flotation (DAF). The F-DAF technique with carrageenan resulted in protein and lipid recoveries from 5% presalting brine of 78 and 38%, respectively. Without flocculation or with only acidification, protein and lipid recoveries in DAF were only 13 and 10%, respectively. Low protein and lipid recoveries, 8–12 and 1.8–8.2%, respectively, were also obtained when spice brine was subjected to only acidification and DAF. The protein content in dry biomasses from 5% presalting brine and spice brine was 36–43 and 13–16%, respectively. The corresponding lipid levels were 23–31 and 9–18%, respectively, with ash levels of 11–20 and 38–45%, respectively. Biomass proteins contained ≤45% essential amino acids, and the lipids had ≤16% long-chain n-3 polyunsaturated fatty acids. Freeze-dried spice brine biomasses were characterized by anchovy- and spice-related sensory attributes. 5% presalting brine biomasses were connected to fish and seafood attributes and showed gel forming capacity. The outlined F-DAF recovery system can thus recover both nutrients and interesting flavors from the herring process waters, which are currently lost from the food chain.

## Abstract 2

Food-grade flocculation and dissolved air flotation for the recovery of protein-enriched biomasses from brines generated during pickling of herring were investigated.

## 1 Introduction

Herring processing is a large industry sector in northern Europe comprising approximately one million tonnes of herring annually, thereby yielding tremendous amounts of process waters during various steps of the value chain (i.e., storage, filleting, presalting, and marination).^1^ Herring process waters contain a variety of macro- and micronutrients, e.g., proteins, peptides, and long-chain n-3 unsaturated polyunsaturated fatty acids (LC n-3 PUFA).^2^ Among the proteins are both salt-soluble myofibrillar proteins and sarcoplasmic proteins such as cathepsins and other enzymes (Szymczak and Kołakowski^2^). When derived from herring marination, waters also contain compounds from the marination formulation, e.g., polyphenols originating from the spices,^3^ salt, and acetic acid.^2c^ Production of pickled herring is performed in three steps: (i) initial short presalting of herring fillets or pieces in a salt brine followed by (ii) storage of the presalted herring in salt-, spice-, or vinegar-containing marination brines for up to 2 years and (iii) removal of the ripening brines and packaging of the marinated herring in small jars with a pickling brine for consumer use {Baron, 2015 #751}. We have estimated that during the presalting and marination steps (i–ii), 100 tonnes of pure proteins and 20 tonnes of pure fatty acids are lost annually when converting 10,000 tonnes of fresh herring into pickled end products (internal communication).

Viewed against the United Nations Sustainable Development Goals (SDGs), seafood companies are today seeking technologies to maximize the use of their raw materials for production of nutritious food, which targets goal No. 2, 3, 12, and 14 (zero hunger, good health and wellbeing, responsible consumption and production, and life below water). Dissolved air flotation (DAF) is a technique based upon the incorporation of air bubbles to adsorb molecules dissolved into water, and it has been extensively used in the wastewater treatment for decades. In seafood-processing companies, DAF is usually proceeded by a nonfood-grade chemical-based pretreatment step to enhance the particle size and improve the floatability of the dissolved compounds.^4^ Studies of chemical-based flocculation/coagulation techniques coupled with DAF for depuration of wastewater report, for instance, on pretreatments with polyacrylamide compounds to remove protein from surimi wash water.^5^ Furthermore, activated carbon combined with DAF was reported to remove the total suspended solid (TSS), chemical oxygen demand (COD), and biological oxygen demand (BOD) from scallop process water.^6^ Furthermore, coagulation with iron salt (FeCl_3_) together with DAF has been used for depuration of fish filleting wastewater.^7^ Baker, Mohamed, Al-Gheethi, and Aziz^8^ studied the depuration of poultry wastewater with DAF and described that the selection of exact pretreatments mostly was dependent upon regulations, costs, and specific characteristics of the wastewater. Using chemical treatments followed by DAF, these authors reported on up to 97% removal of the poultry wastewater COD. In the study of Karnena, Konni, Dwarapureddi, and Saritha,^9^ depuration of fish processing water using coagulation/flocculation alone provided 92% COD reduction using a combination of Tanfloc, FeCl_3_, and Zetag. Although the mentioned studies were successful in wastewater depuration, the use of nonfood-grade chemicals limited the application of the recovered sludge to feedstock for biogas production purposes. To widen applications also to food/feed, alternatives to DAF must be used, or an initial treatment before DAF must be done with a food/feed-grade coagulant/flocculant. Regarding the former, pressure-driven membranes for microfiltration, ultrafiltration, nanofiltration, and reverse osmosis have for instance been used to recover an astaxanthin-rich fraction from shrimp cooking water^10^ or proteins from poultry processing water,^11^ soy protein isolation process water,^12^ or cheddar cheese effluents.^13^ Membrane processes do not require pretreatments with flocculants/coagulants but inevitably entail fouling, which is a major obstacle to reach efficiency in the separation.^14^

Regarding coupling of the DAF technique with food-grade flocculation (F), a few studies have been published on aquaculture wastewater,^15^ and we earlier reported on pilot-scale recovery of a protein-rich biomass from shrimp processing water using alginate and carrageenan combined with DAF.^16^ The protein yield obtained was up to 97%, and after spray drying, the biomass contained 61% protein and 18% lipids (Forghani, Sørensen, Johannesson, Svendsen, and Undeland^16^). However, to the best of our knowledge, there is no report on pilot-scale biomass recovery from herring industry process waters using F-DAF, although this could provide a low-cost and easily implementable solution to recover currently lost nutrients, thereby contributing to several SDGs via a circular approach.

In the present study, our aim was to investigate the recovery of proteins and lipids present in herring pickling process waters using the F-DAF technique. We investigated the effect of acidification, without and with carrageenan, to flocculate a 5% presalting brine derived in primary processing. For a spice brine derived from herring ripening in barrels, only acidification was used since the high salt content prevented the use of flocculants. Acidification was done here with HCl or a combination of vinegar brine and HCl, to increase the sustainability of the process. After freeze drying, the recovered biomasses were subjected to analyses of crude composition, fatty acid and amino acid profiles, gel forming capacity, and sensory characteristics.

## 2 Materials and Methods

### 2.1 Materials

In this study, 5% presalting brine from a primary producer (Sweden Pelagic AB) as well as spice brine and vinegar brines from a secondary producer (Klädesholmen seafood AB), all three emerging as side streams, was used as raw materials for the F-DAF trials during October 2019. Hydrochloric acid (30%) for acidification of the selected process waters was obtained from Nitor, Sweden, and λ-carrageenan (Viscarine GP-109NF) was provided by FMC Food and Nutrition (PA).

### 2.2 Methods

#### 2.2.1 F-DAF Treatment of Process Waters

**Figure 1:**
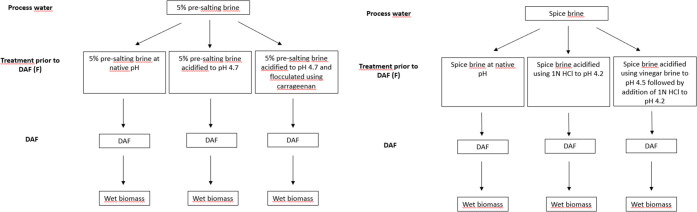
Schematic diagram over the study design including F-DAF.

Flocculation of process waters was carried out on-site at the companies. In brief, 600–700 L of brine was treated according to an in-house method established on a lab scale. Stock solution (1% w/v) of carrageenan was made by constant mixing of carrageenan powder in 60 °C tap water, after which the solution was cooled to 10 °C. In brief, the pH of the process water (5% presalting brine, 600–700 L per batch) was first adjusted to 4.7 using 1 N HCl; thereafter, flocculation was achieved during constant stirring by adding 1% carrageenan stock solution to a final concentration of 0.45 g/L. During flocculation, the temperature remained below 10 °C. For 5% presalting brine, three F-DAF runs were performed, including (i) a control run with 5% presalting brine at native pH (6.5) (referred to as B), (ii) 5% presalting brine acidified to pH 4.7 for isoelectric precipitation (referred to as BP), and (iii) 5% presalting brine acidified to pH 4.7 and flocculated using carrageenan (referred to as BC). For the spice brine, three F-DAF runs comprised (i) a control run at native pH (5.8) (referred to as SB), (ii) a run with the brine acidified using 1 N HCl to pH 4.2 to conduct isoelectric precipitation (referred to as SBP), and (iii) one run where the brine was acidified using vinegar brine to pH 4.5 followed by addition of 1 N HCl to pH 4.2 (referred to as SBV); throughout the paper, these abbreviations are used when referring to a biomass originating from brine treated with F-DAF (Figure 1). Throughout the text, “5% presalting brine” and “spice brine” are used when referring to raw brines prior to treatment with F-DAF. Carrageenan was left out for the spice brine due to its high salt content, which on the lab scale was found to hamper flocculation. The treated waters were pumped at 300 L/h into the flotation unit (see further info below), while micro-air bubbles were generated and injected into the incoming water. The generation of microbubbles continued for 20 min after the inlet pumping was ceased. Collection of biomasses was initiated once a thick foam layer was formed on top of the water in the flotation unit and continued regularly until the end of the air bubbling period. The temperature of process waters was below 10 °C during the entire F-DAF treatment. For each run, the weight of the total collected biomass was recorded, and samples from the inlet and biomass as well as the outlet were collected and stored at −80 °C until further analyses. The crude compositions (protein, lipid, ash, and moisture contents) of inlets and biomasses were analyzed, and the recovery of protein and lipid during each run was determined. Flotation was performed immediately after flocculation using a 376 L rectangular flotation unit produced in 304 L stainless steel (Bio-Aqua A/S, Denmark) and equipped with a pneumatic scraper with a rubber blade.^16^

#### 2.2.2 Analytical Methods

##### 2.2.2.1 Proximate Composition

The protein content of the process waters (inlets), outlets, and wet biomasses was measured following the method of Lowry, Rosebrough, Farr, and Randall^17^ modified by Markwell, Haas, Bieber, and Tolbert^18^ using serum bovine albumin as a standard in the concentration range of 10–110 μg/mL. Prior to analysis, biomasses were mixed with a 1 N NaOH solution to dissolve nondissolved proteins. The Lowry method only captures proteins/peptides and not amino acids since it is based on the interaction of copper with the peptide bond. Some of the wet biomasses were also freeze-dried using a Labconco freeze drier at below 0.02 mBar with the temperature of the collection chamber being −50 °C. Crude protein of the freeze-dried biomasses was analyzed via the Dumas method using a nitrogen analyzer (LECO TruMac N, MI) with a conversion factor of 5.58.^19^ The lipid content was analyzed gravimetrically according to the method described by Lee, Trevino, and Chaiyawat.^20^ Protein and lipid recoveries were calculated using the following formula: . Dry matter and ash contents were measured gravimetrically after drying the samples at 105 and 550 °C, respectively, for 24 and 3 h.

##### 2.2.2.2 Amino Acid Analysis

Amino acids were analyzed following the method previously described by Özcan and Şenyuva.^21^ 40 mg of freeze-dried biomass was mixed with 4 mL of 6N HCl and flushed with nitrogen gas for 30 s; the tubes were maintained at 110 °C for 24 h to complete the acid hydrolysis, after which the samples were filtered, diluted, and measured by LC/APCI-MS. 2 μL samples were injected into the LC system (Agilent 1100 HPLC, Waldbron, Germany), and separation was carried out on a Phenomenex column (C18 (2) 250 μm × 4.6 μm × 3 μm), coupled to an Agilent 6120 quadrupole in the SIM positive mode (Agilent Technologies, Germany). The peaks were compared and quantified against an amino acid standard mix (ref# NCI0180. 20088, Thermo Scientific Pierce).

##### 2.2.2.3 Fatty Acid Analysis

Fatty acids in the freeze-dried biomasses were measured following lipid extraction with chloroform and methanol (2:1) Lee, Trevino, and Chaiyawat.^20^ C:17 was added as an internal standard prior to methylation and vortexed for 10 s, and finally, 0.5% NaCl at 1:2.75 *v*/*v* ratio was added. Following phase separation, chloroform was recovered and evaporated at 40 °C. Methylation was conducted according to Lepage and Roy^22^ with minor modifications. 2 mL of toluene and 2 mL of acetylchloride:methanol (10%) were added to the extracted lipids, and the solution was incubated at 60 °C for 120 min. 1 mL of Milli-Q water and 2 mL of petroleum ether were added to each glass test tube, which were vortexed for 10 s and centrifuged at 2500*g* for 5 min. The upper phase was transferred to a new glass test tube and evaporated under nitrogen at 40 °C. The evaporated samples were dissolved in 0.5 mL of isooctane. Identification and quantification of fatty acids were carried out by GS-MS using an Agilent technologies 7890 A GC system connected to an Agilent technologies 5975 inert MSD (Kista, Sweden) as described elsewhere.^23^ Total fatty acids were calculated as the sum of all measured fatty acids in the sample minus the internal standard.

#### 2.2.3 Gel Preparation Using Freeze-Dried Biomasses Recovered from 5% Brine

Gel preparation from freeze-dried biomasses recovered from 5% presalting brine was performed as previously described Abdollahi, Rezaei, Jafarpour and Undeland^24^ with minor modifications. In brief, 40 g of gel was prepared by weighing enough amount of each powder and Milli-Q water to reach 10% protein content. 1 N NaOH was used to adjust the pH to 7 in a chopper on ice. Spice brine-derived biomasses were not evaluated due to the presence of spice particles that interfered with the gel formation.

#### 2.2.4 Dynamic Oscillatory Rheological Analyses of Freeze-Dried Biomasses Recovered from 5% Brine

Dynamic viscoelastic properties of the freeze-dried biomasses were measured using parallel-plate geometry (25 mm plate diameter and 1 mm plate gap) mounted on a dynamic rheometer (Paar Physica Rheometer MCR 300, Anton Paar GmbH, Austria), operated in an oscillating mode. 1–2 g of sample paste (Section 2.2.3) was placed over the plate, and once the sample was pressed by lowering the upper plate, the excess sample was removed. The exposed sample perimeter was covered with inorganic oil to prevent evaporation. The storage modulus (*G*′), loss modulus (*G*″), and phase angle (δ) were recorded during a temperature increase from 20 to 90 C at 1 °C/min heating rate.^24,25^ The temperature sweep was conducted at 1% constant strain and 0.1 Hz frequency in the linear viscoelasticity region. The applied stress (25 Pa) was also in the linear viscoelasticity region of the tested gel samples according to the primarily conducted stress sweep test. The rheogram data are reported as the mean value of three independent experiments.

#### 2.2.5 Polypeptide Profiling Using Sodium Dodecyl Sulfate–Polyacrylamide gel electrophoresis (SDS-PAGE)

The polypeptide pattern of biomasses was visualized using SDS-PAGE^26^ using precast minigradient gels 4–20% (Bio-Rad). Samples were prepared by adding 27 mL of SDS solution (5%) to 3 g of wet samples or 0.3 g of freeze-dried samples, after which they were heated to 85 °C for 1 h to dissolve the protein. Samples were centrifuged at 5000*g* for 20 min to remove the undissolved residuals, and the supernatant was diluted to 4 g/L protein, mixed with an equal amount of Laemmli buffer (Bio-Rad), and boiled for 5 min. 20 μg of protein was loaded into each lane, and the polypeptide molecular standard was in a broad range (prestained dual color standard, 10–250 kDa, Bio-Rad). Protein bands were stained using Coomassie brilliant blue G-250. The gel was scanned in a Geldoc Go imaging system (Bio-Rad), and the picture was analyzed using Image Lab 6.1 (Bio-Rad).

#### 2.2.6 Sensory Analysis

Sensory analysis was done at the Perception-lab Gothenburg (equipped accord. ISO 8589:2007) at the RISE Research Institutes of Sweden AB. Samples were profiled using the free sorting task. The sample consisted of powders of different biomasses (B, BP, BC, SB, SBP, and SBV), which were dissolved in water at two concentrations, low (L): 3% (dry weight (dw)/wet weight (ww)) and high (H): 5% (dw/ww). Thirteen trained panelists evaluated a total of 12 samples in duplicate during one session with 10 min break between the duplicates. The exercise consisted of grouping the samples based upon similarities in taste and flavor. Panelists smelled and tasted the sample and grouped them based on similarities. Participants could form as many groups as they preferred, if one group was represented by at least one sample. A list of attributes (in Swedish) was provided, and the panelists were asked to provide 3–5 attributes from the attribute list to describe their groups.

The list of attributes was created using the consensus method with four highly trained panelists. For a 2 h session, the panelists tasted samples, which covered the sensory space of the evaluated samples, and after reaching consensus, they built a list of attributes relevant for the study. The list of attributes consisted of the following: anchovy (smell and taste), anise (smell and taste), Christmas spices (smell), cloves (smell), dill (taste), fatty fish (smell and taste), fish innards (taste), fish liver oil (smell), fish soup (smell), fish soup (taste), fishing port (smell), fish oil (smell), fishy (smell and taste), grassy (smell), Kalles kaviar (smokey) (smell and taste), Kalles kaviar (smokey) (taste), pimento (smell), mackerel (smell) makrillspad (taste and smell), matjessill (taste) mild taste; rancid (smell and taste), raw fish (smell and taste), raw white fish (smell and taste), rubbery smell, salt (low, medium, and high), sea water/seaweed smell, herring smell, seafood taste, shrimp smell, herring (smell and taste), smokey (smell), solvent (smell), sour taste, spicy (smell), stale (taste), sweet taste, sweet-like smell, unfrozen fish cubes (smell), and watery (taste).

The samples were blind-coded with three-digit codes and presented to the panelists in a randomized order. All samples were served in ISO black glasses at room temperature. 30 mL of each sample was served for the evaluation, and water and crackers were provided to neutralize the taste buds between the different samples. Panelists could retaste any samples as many times as they needed, although they were advised that retasting could lead to confusion.^27^

The study was conducted in compliance with the Declaration of Helsinki,^28^ and all participants provided written consent before participation and were recompensated economically upon the completion of sensory analyses. The study was assessed for compliance with national research requirements through an internal process at the RISE Research Institutes of Sweden and was approved by managers at the Department of Material and Surface Design. Personal data were collected and handled in compliance with the General Data Protection Regulation (EU) 2016/679 (GDPR).

#### 2.2.7 Statistical Analysis

One-way analysis of variance (ANOVA) and Tukey test were used to determine the significant differences between the tested variables. In the case of mass balance analyses, only biomass data were subjected to ANOVA. Differences with a probability value of <0.05 were considered significant, and all data were reported in the form of mean ± SD. All analyses were carried out in duplicates (*n* = 2), except for the protein content and dynamic rheology, which were done in triplicates (*n* = 3).

The statistical evaluation of the sensory results was performed using XLStat (Addinsoft, New York, NY). A co-occurrence matrix was constructed using the frequency of each possible pair of samples as placed together in the same group, and the matrix was further analyzed using multidimensional scaling (MDS) to build the similarity space. Furthermore, a contingency table of the attributes used to describe the groups was also constructed. The contingency table was used to tune a correspondence analysis (CA) to understand the sensory space of the similarity space. Hierarchical cluster analysis (HCA) was also used as an agglomerative strategy to understand the similarities between the products. HCA was applied following the Ward criterion on the factorial coordinates of the samples in the space defined by the correspondence analysis. Data were captured using EyeQuestion R. (v.3.8.6, Logic 8 BV software, Netherlands).

## 3 Results and Discussion

### 3.1 Capacity of F-DAF Treatment to Concentrate Protein and Lipid

Protein flocculation using different polysaccharides is a well-described phenomenon. In the DAF technique, suspended macronutrients attach to air bubbles, which float/move toward the surface due to the changes in density. Results showed that the protein and lipid recoveries obtained during treatment of 5% herring salt brine with DAF were significantly affected (*P* < 0.05) by preflocculation treatment with carrageenan (Table 1). Acidification together with carrageenan flocculation (BC samples) gave the highest recoveries of proteins (78%) and lipids (38%). However, for control runs with presalting brine (B) and acid-precipitated brine (BP), DAF performed equally low and yielded protein and lipid recoveries of only 13 and 10%, respectively. We recently reported that flocculation of acidified shrimp processing waters with either alginate or carrageenan together with DAF provided 27–97% protein recovery.^16^ In the present study, combining DAF with carrageenan flocculation was also the most effective method to upconcentrate protein, with fourfold higher protein in the recovered BC-biomass compared to that in the inlet brine. In BP biomass and B biomass, the concentration factors were 3.4-fold and 2.7-fold, respectively. Protein and moisture contents in BP and BC samples were 7.5 and 8.1% (ww) and 90.5–90.8%, respectively. Thus, although the protein recovery was low, treatment with only acidification significantly (*P* < 0.05) upconcentrated protein compared to that in the control (B), which was subjected to DAF at the native pH of 6.5. A reason for the low protein recovery without a flocculant could be the small particle sizes. In the presence of carrageenan, larger flocs are built, which affect their rate of migration to the surface, providing a faster recovery process.^4^ The ash content of B, BP, and BC biomasses ranged between 0.9 and 1.8% and was significantly different (*P* < 0.05) between B and BC.

**Table 1:** Crude Composition Including Protein Content, Lipid Content, Moisture Content, and Ash Content (Wet Weight (ww) Basis) as well as Protein and Lipid Recovery upon Treating of 5% Presalting Brine without/with Acidification and Carrageenan^a^^,^^b^^,^^c^

process water	fraction	protein content (%)	lipid content (%)	moisture content (%)	ash content (%)	protein recovery (%)	lipid recovery (%)
B	inlet	1.8 ± 0.0	0.8 ± 0.1	94.2 ± 2.0			
	biomass	5.1 ± 0.1^B^	1.7 ± 0.3^B^	94.1 ± 3.0^B^	0.9 ± 0.0^B^	13.4	10.1
	outlet	1.0 ± 0.0	ND	ND			
BP	inlet	2.19 ± 0.0	1.0 ± 0.0	94.4 ± 1.0			
	biomass	7.49 ± 0.1^A^	2.6 ± 0.1^A^	90.5 ± 0.9^A^	1.6 ± 0.2^AB^	13.2	10.5
	outlet	1.44 ± 0.0	ND	ND			
BC	inlet	1.96 ± 0.0	1.1 ± 0.1	94.2 ± 0.8			
	biomass	8.11 ± 0.1^A^	2.2 ± 0.1^AB^	90.8 ± 0.3^A^	1.8 ± 0.1^A^	77.9	38.2
	outlet	0.72 ± 0.0	ND	ND			

a Analytical data are shown as mean ± SD (*n* = 2).

b ND. not determined.

c Means with different superscript letters within the same column indicate significant differences among biomasses (*P* < 0.05). B, 5% presalting brine at native pH (6.5); BP, 5% presalting brine acidified to pH 4.7; and BC, 5% presalting brine acidified to pH 4.7 and flocculated using carrageenan.

For treatments with spice brine, acid-induced precipitation with HCl prior to DAF (SBP) improved the protein recovery to 12% compared to running DAF at the native pH of 5.8 (2.3% protein recovery). Furthermore, the use of vinegar brine to replace portions of the HCl (SBV) reduced the protein recovery down to 8.1% (Table 2). Similarly, lipid recovery was improved up to 4.5-fold by acidification; the recoveries for SBP and SBV were 8.2 and 7.0%, respectively. Overall, our data suggest that acidification is a crucial step to maximize protein and lipid recoveries within DAF, but when possible, it should be combined with flocculants such as carrageenan.

**Table 2:** Crude Composition Including Protein Content, Lipid Content, and Dry Matter and Ash Content (ww Basis) as well as Protein and Lipid Recovery upon Treatment of Spice Brine without/with Acidification Using HCl or Vinegar Brine Together with HCl^a^^,^^b^^,^^c^

process water	fraction	protein content (%)	lipid content (%)	moisture content (%)	ash content (%)	protein recovery (%)	lipid recovery (%)
SB	inlet	3.0 ± 0.0	4.9 ± 0.7	75.0 ± 2.5			
	biomass	4.8 ± 0.0^C^	6.1 ± 0.4^A^	78.5 ± 1.7^A^	6.2 ± 0.0^A^	2.3	1.8
	outlet	1.5 ± 0.0	ND	ND			
SBP	inlet	2.4 ± 0.2	1.0 ± 0.1	79.2 ± 1.2			
	biomass	7.3 ± 0.0^A^	2.0 ± 0.1^B^	80.4 ± 2.0^C^	12.8 ± 3.9^A^	12.1	8.2
	outlet	2.2 ± 0.0	ND	ND			
SBV	inlet	2.4 ± 0.0	0.8 ± 0.0	78.7 ± 1.1			
	biomass	6.5 ± 0.1^B^	1.9 ± 0.2^B^	79.4 ± 2.1^B^	10.2 ± 0.0^A^	8.1	7.0
	outlet	2.1 ± 0.2	ND	ND			

a Analytical data are shown as mean ± SD (*n* = 2).

b ND. not determined.

c Means with different superscript letters within the same column indicate significant differences among biomasses (*P* < 0.05). SB, spice brine at native pH (5.8); SBP, spice brine acidified using 1 N HCl to pH 4.2; and SBV, spice brine acidified using vinegar brine to pH 4.5 followed by addition of 1 N HCl to pH 4.2.

### 3.2 Characteristics of Freeze-Dried Biomasses

Biomasses recovered from F-DAF were freeze-dried, and the crude composition as well as the amino acid profile and fatty acid profile of powders is reported in Tables 3–5. Powders recovered from 5% presalting brine contained 36–43% protein, 23–31% lipids, and 12–20% ash (Table 3). The highest levels of protein and ash were identified in BC, while lipids were at the highest in B. For powders recovered from spice brine, the ash content was the major constituent (38–45%), reflecting their high salt content. Their protein content ranged between 13 and 16% with that of lipids between 10 and 18% (Table 3). The highest lipid content (18%) was found in dried biomasses recovered at native pH (SB), while protein was 16% in both SBP and SBV. Commercial fish meal typically contains 55–70% protein, 8–11% fat, and 17–24% ash.^29^ The BC powder in our study had a higher lipid content (23%) but a slightly lower protein level (43%), which reveals its potential as an aquafeed ingredient.

**Table 3:** Proximate Composition (%) of Freeze-Dried Biomasses Recovered from 5% Presalting Brine without/with Acidification and Carrageenan and Treatment of Spice Brine without/with Acidification Using HCl or Vinegar Brine Together with HCl^a^^,^^b^

freeze-dried biomass	protein content (%)	lipid content (%)	ash content (%)	moisture content (%)
B	36.0 ± 0.4^C^	31.0 ± 1.0^A^	11.8 ± 0.4^B^	2.1 ± 0.1^A^
BP	38.4 ± 0.5^B^	28.4 ± 0.5^B^	14.4 ± 0.3^B^	2.0 ± 0.1^A^
BC	43.5 ± 0.5^A^	23.0 ± 1.6^C^	20.5 ± 2.0^A^	2.1 ± 0.0^A^
SB	13.7 ± 0.1^B^	18.0 ± 0.2^A^	38.0 ± 0.3^C^	0.4 ± 0.0^C^
SBP	16.5 ± 0.2^A^	11.6 ± 0.3^B^	42.4 ± 0.0^B^	3.3 ± 0.0^B^
SBV	16.5 ± 0.1^A^	9.7 ± 1.7^B^	45.0 ± 0.0^A^	2.2 ± 0.0^A^

a DAF was performed as downstream separation to recover biomasses for all the samples.

b Means with different superscript letters within the same column for each water type indicate significant differences among these samples (*p* < 0.05). B, 5% presalting brine at native pH (6.5); BP, 5% presalting brine acidified to pH 4.7; BC, 5% presalting brine acidified to pH 4.7 and flocculated using carrageenan; SB, spice brine at native pH (5.8); SBP, spice brine acidified using 1 N HCl to pH 4.2; and SBV, spice brine acidified using vinegar brine to pH 4.5 followed by addition of 1 N HCl to pH 4.2.

The amino acid content of freeze-dried biomasses recovered from 5% presalting brine and spice brine is presented in Table 4. In all biomasses, GLX was the predominant amino acid followed by ASP, LYS, and LEU, and all these amino acids were between 25 and 46 mg/g dw when recovered from B, BC, and BP, with the corresponding values being 9–19 mg/g dw for biomasses recovered from SB, SBP, and SBV. The lower values of the above-stated amino acids in biomasses recovered from SB compared to B correspond to their lower protein content. Of the EAAs, LYS and LEU were predominant followed by THR and VAL. EAAs for humans comprise LYS, HIS, THR, VAL, MET, ILE, LEU, and PHE, and these AA constituted 44–45% of the TAA in all biomasses, which is close to the levels reported for beef and fish muscles, 47 and 45%, respectively.^30^ The biomasses recovered in our study therefore exhibited a good balance of EAAs for human consumption provided that the salt content is within the permitted level during consumption. Furthermore, the contents of individual EAAs in our study were far above the requirement for human nutrition,^31^ despite the fact that MET and HIS were underestimated due to susceptibility to acid hydrolysis. For fish, the EAAs are the same as those listed for humans except for THR, which is replaced by ARG, and in our study, the EAA constituted 44% of the TAA.

**Table 4:** Amino Acid Content (mg/g) of Freeze-Dried Biomasses Recovered from Treatment of 5% Presalting Brine without/with Acidification and Carrageenan and Treatment of Spice Brine without/with Acidification Using HCl or Vinegar Brine Together with HCl^a^^,^^b^

	amino acid (mg/g)						
	B	BP	BC	SB	SBP	SBV	FAO/WHO adult (mg/g protein)
LYS^c^	31.3 ± 5.2	31.3 ± 0.6	34.6 ± 0.2	11.8 ± 0.2^C^	14.2 ± 0.2^B^	15.2 ± 0.2^A^	45
ARG	13.2 ± 2.4	13.2 ± 0.5	13.7 ± 0.1	3.0 ± 0.1^B^	3.4 ± 0.0^A^	3.3 ± 0.1^AB^	
HIS^c^	6.8 ± 1.0^B^	8.0 ± 0.3^AB^	9.5 ± 0.5^A^	1.9 ± 0.0^B^	2.1 ± 0.0^A^	2.1 ± 0.0^A^	15
GLY	11.8 ± 1.8	13.2 ± 0.9	15.5 ± 1.3	5.5 ± 0.0^A^	5.5 ± 0.0^A^	5.5 ± 0.1^A^	
SER	14.1 ± 2.3	14.5 ± 0.2	16.4 ± 0.5	5.7 ±0.0^B^	6.4 ± 0.2^A^	6.5 ± 0.0^A^	
ALA	17.8 ± 2.7	18.8 ± 0.4	22.3 ± 0.8	7.5 ± 0.2^B^	8.6 ± 0.2^A^	9.0 ± 0.0^A^	
THR^c^	15.2 ± 2.6	16.5 ± 0.0	18.6 ± 0.8	6.3 ± 0.0^B^	7.1 ± 0.0^A^	7.4 ± 0.1^A^	23
GLX	45.3 ± 8.8	44.8 ± 0.7	46.8 ± 0.9	15.2 ± 0.2^C^	18.4 ± 0.2^B^	19.5 ± 0.2^A^	
ASX	30.9 ± 4.9	32.4 ± 0.7	35.8 ± 1.8	10.9 ± 0.1^B^	12.4 ± 0.2^A^	13.0 ± 0.2^A^	
PRO	11.2 ± 1.7	11.9 ± 0.1	13.3 ± 0.5	4.3 ± 0.0^A^	4.4 ± 0.0^A^	4.5 ± 0.0^A^	
VAL^c^	13.3 ± 2.7	14.9 ± 0.9	16.5 ± 0.4	5.4 ± 0.2^B^	6.0 ± 0.0^AB^	6.1 ± 0.1^A^	39
MET^c^	10.8 ± 2.0	10.3 ± 1.9	10.4 ± 2.2	2.9 ± 0.8^A^	3.8 ± 0.0^A^	3.8 ± 0.1^A^	17
TYR	12.1 ± 1.6	11.9 ± 0.2	13.6 ± 0.4	4.5 ± 0.1^B^	4.9 ± 0.0^A^	4.6 ± 0.1^AB^	
ILE^c^	11.5 ± 2.7	12.4 ± 0.7	14.0 ± 0.7	4.8 ± 0.2^A^	5.3 ± 0.2^A^	5.2 ± 0.2^A^	30
LEU^c^	25.5 ± 5.0	25.9 ± 0.5	28.9 ± 1.0	8.9 ± 0.2^B^	10.5 ± 0.1^A^	10.5 ± 0.5^A^	59
PHE^c^	11.5 ± 2.5	13.1 ± 0.1	14.6 ± 0.4	4.3 ± 0.0^B^	4.6 ± 0.1^A^	4.7 ± 0.0^A^	19
TAA	283.1	293.8	325.3	102.9	118.2	121.2	
TEAA	125.9	132.4	147.2	46.3	53.8	55.0	
TEAA/TAA	0.44	0.45	0.45	0.45	0.45	0.45	

a DAF was performed as downstream separation to recover biomasses for all the samples.

b Means with different superscript letters within the same row for SB, SBP, and SBV indicate significant differences among these samples (*p* < 0.05). For B, BP, and BC, the superscript letters were only included for HIS, as there was no significant differences for the rest of the amino acids (*p* ≤ 0.05). GLX, GLU + GLN; ASX, ASP + ASN; TAA, total amino acid; TEAA, total essential amino acid; and TNEAA, total nonessential amino acid.

c EAA to human. B, 5% presalting brine at native pH (6.5); BP, 5% presalting brine acidified to pH 4.7; BC, 5% presalting brine acidified to pH 4.7 and flocculated using carrageenan; SB, spice brine at native pH (5.8); SBP, spice brine acidified using 1 N HCl to pH 4.2; and SBV, spice brine acidified using vinegar brine to pH 4.5 followed by addition of 1 N HCl to pH 4.2.

The total fatty acid content in freeze-dried biomasses derived from 5% presalting brine ranged between 165.7 and 227.6 mg/g (Table 5), whereas in dried biomasses derived from spice brine, the range was 10–45 mg/g. In all the biomasses derived from 5% presalting brine, C16:0 was the most abundant fatty acid (31–44 mg/g) followed by C20:1 and C16:1, which ranged between 10 and 44 mg/g. These observations were also relevant for biomasses derived from spice brine. The levels of long-chain (LC) n-3 polyunsaturated fatty acids (PUFAs) (C 20:5 and C22:6) in the dried biomasses were 31–46 mg/g (Table 5). Overall, monounsaturated fatty acids (MUFA) were predominant (54–64%) in dried biomasses from both brines, followed by PUFAs (20–24%) and then saturated fatty acids (SFA) (15–20%). In herring muscles, 44–46% MUFAs, 20–32% PUFAs, and 17–21% SFAs have been earlier reported,^32^ which agreed well with our data, although the n-3 to n-6 ratio of herring muscles was slightly higher (9: 11.5) compared to that in biomasses recovered from the two brines (7.4: 8.2). The presence of C20:1, C12:1, and C13:1 could be due to the contamination occurred during downstream treatment of herring brines. Overall, the pretreatments prior to DAF did not have a significant effect (*p* < 0.05) on the fatty acid profiles of the biomasses recovered from 5% presalting brine.

**Table 5:** Fatty Acid Profile (mg/g) of Freeze-Dried Biomasses Recovered from Treatment of 5% Presalting Brine without/with Acidification and Carrageenan as well as Freeze-Dried Biomasses Recovered from Treatment of Spice Brine without/with Acidification Using HCl or Vinegar Brine Together with HCl^a^^,^^b^

fatty acid (mg/g)	B	BP	BC	SB	SBP	SBV
C12:0	0.24 ± 0.09	0.21 ± 0.03	0.17 ± 0.03	0.04 ± 0.00^A^	0.06 ± 0.03^A^	0.08± 0.01^A^
C15:0	1.47 ± 0.59	1.28 ± 0.29	1.01 ± 0.24	0.20 ± 0.00^B^	0.25 ± 0.12^B^	0.34 ± 0.01^A^
C16:0	44.6 ± 17.7	39.79 ± 8.74	31.5 ± 6.50	6.58 ± 0.19^A^	8.88 ± 4.18^A^	11.4 ± 0.1^A^
C19:0	0.11 ± 0.05	0.07 ± 0.03	0.08 ± 0.02	0.01 ± 0.00^A^	0.02 ± 0.01^A^	0.02 ± 0.01^A^
C12:1	28.2 ± 3.0	28.40 ± 0.12	27.5 ± 3.2	11.5 ± 1.3^A^	15.1 ± 7.1^A^	15.1 ± 2.0^A^
C13:1	27.3 ± 10.8	24.59 ± 5.42	19.2± 3.8	3.88 ± 0.07^A^	5.3 ± 2.5^A^	7.3 ± 0.2^A^
C16:1 (n-7)	15.5 ± 6.2	13.86 ± 3.06	10.8 ± 2.2	2.89 ± 0.07^A^	3.85± 1.8^A^	5.2 ± 0.1^A^
C17:1 (n-7)	3.3 ± 1.3	2.91 ± 0.70	2.30 ± 0.54	0.49 ± 0.01^B^	0.65 ± 0.30^AB^	0.8 ± 0.14^A^
C18:1 (n-9, n-12)	3.96 ± 1.58	3.55 ± 0.89	2.82 ± 0.61	0.81 ± 0.01^A^	1.10 ± 0.52^A^	1.3 ± 0.00^A^
C20:1 (n-13, n-15)	3.10 ± 1.42	2.48 ± 0.54	2.03 ± 0.46	0.56 ± 0.00^AB^	0.75 ± 0.35^A^	0.96 ± 0.01^B^
C20:1 (n-9)	37.8 ± 15.5	33.6 ± 8.1	26.2 ± 6.2	8.01 ± 0.30^AB^	10.6 ± 5.0^A^	13.4 ± 0.1^B^
C22:1 (n-9)	3.79 ± 1.25	3.21 ± 0.96	2.59 ± 0.76	0.85 ± 0.29^A^	2.01 ± 0.94^A^	1.86 ± 0.36^A^
C24:1 (n-9)	1.49 ± 0.52	1.16 ± 0.33	1.00 ± 0.31	0.16 ± 0.05^B^	0.15 ± 0.07^B^	0.24 ± 0.09^A^
C18:2 (n-6)	4.57 ± 1.89^B^	3.99 ± 0.92^B^	3.11 ± 0.71^A^	0.73 ± 0.00^B^	0.99 ± 0.46^B^	1.32 ± 0.04^A^
C20:2 (n-6)	0.70 ± 0.12	0.66 ± 0.07	0.46 ± 0.02	0.08 ± 0.01^B^	0.09 ± 0.04^AB^	0.23 ± 0.02^B^
C18:3 (n-6)	0.52 ± 0.21	0.42 ± 0.11	0.34 ± 0.05	0.06 ± 0.01^B^	0.08 ± 0.04^AB^	0.15 ± 0.00^A^
C20:4 (n-6)	0.92 ± 0.35	0.70 ± 0.16	0.58 ± 0.19	0.11 ± 0.01^A^	0.16 ± 0.07^A^	0.23 ± 0.05^A^
C18:3 (n-3)	3.74 ± 1.51	3.18 ± 0.80	2.53 ± 0.45	0.53 ± 0.00^B^	0.73 ± 0.34^B^	1.03 ± 0.04^A^
C20:3 (n-3)	0.42 ± 0.08	0.31 ± 0.12	0.27 ± 0.04	0.02 ± 0.01^B^	0.03 ± 0.01^B^	0.06 ± 0.00^A^
C20:5 (n-3)	20.5 ± 8.6	18.2 ± 4.4	14.1 ± 3.3	3.81 ± 0.11^AB^	5.18 ± 2.44^A^	6.96 ± 0.02^B^
C22:5 (n-3)	1.89 ± 0.83	1.58 ± 0.65	1.18 ± 0.57	0.25 ± 0.01^B^	0.36 ± 0.17^B^	0.44 ± 0.02^A^
C22:6 (n-3)	23.3 ± 9.5	19.8 ± 4.97	15.6 ± 3.8	3.37 ± 0.06^AB^	4.63 ± 2.13^A^	6.15 ± 0.5^B^
% SFA	20.4	20.3	19.8	15.1	15.0	15.9
% MUFA	54.7	55.8	57.0	64.9	64.8	62.0
% PUFA	24.9	24.0	23.1	20.0	20.1	22.1
% n-3	21.9	21.1	20.4	17.8	17.9	19.6
% LC n-3	20.3	19.6	18.9	16.6	16.7	18.2
% n-6	2.9	2.8	2.7	2.2	2.2	2.6
n-3:n-6	7.44	7.45	7.51	8.0	8.2	7.6

a DAF was performed as downstream separation to recover biomasses for all the samples.

b For B, BP and BC, no significant differences (*p* < 0.05) were found for the reported fatty acids except for C18:2 (n-6). Means with different superscript letters within the same row for SB, SBP, and SBV indicate significant differences among these samples (*p* < 0.05). B, 5% presalting brine at native pH (6.5); BP, 5% presalting brine acidified to pH 4.7; BC, 5% presalting brine acidified to pH 4.7 and flocculated using carrageenan; SB, spice brine at native pH (5.8); SBP, spice brine acidified using 1 N HCl to pH 4.2; and SBV, spice brine acidified using vinegar brine to pH 4.5 followed by addition of 1 N HCl to pH 4.2.

### 3.3 Polypeptide Profiling by SDS-PAGE

**Figure 2:**
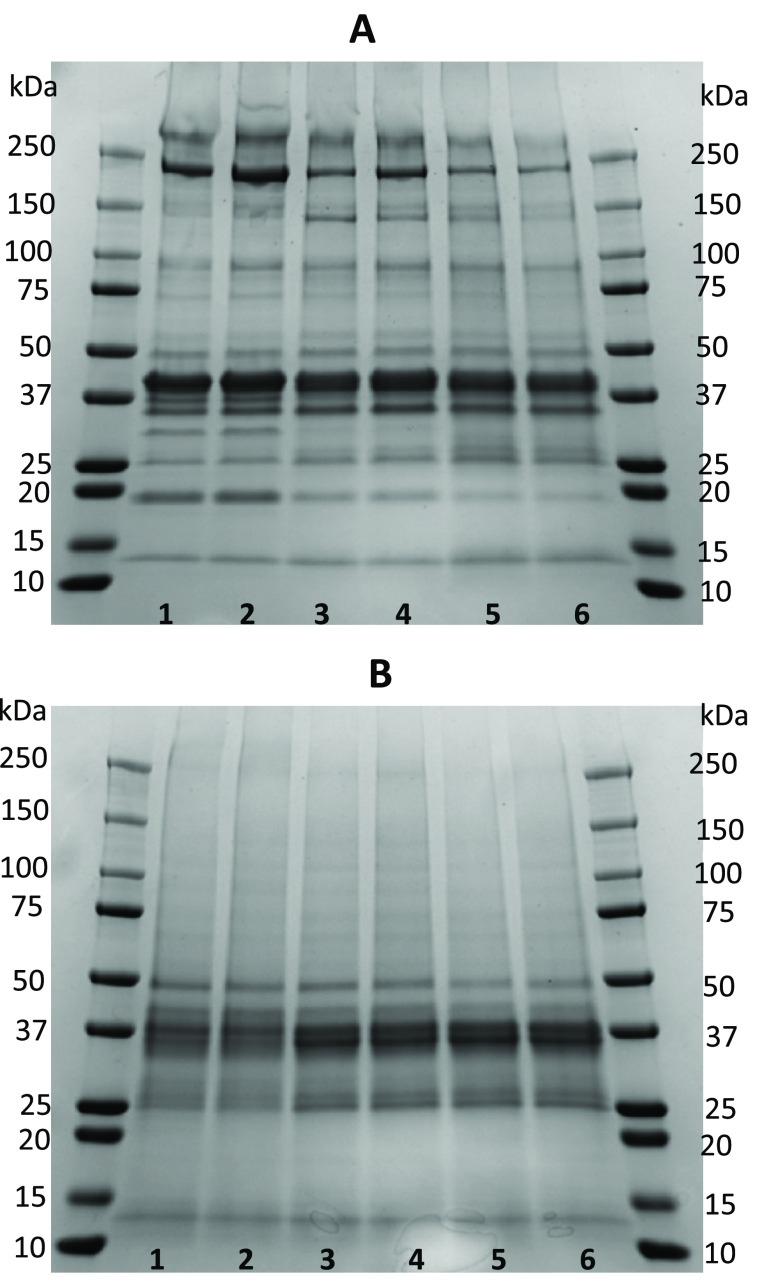
Polypeptide profiling of biomass recovered from 5% presalting brine and spice brine upon F-DAF. Gel A: (1) B-wet biomass; (2) B-dried biomass; (3). BP-wet biomass; (4) BP-dried biomass; (5) BC-wet biomass, and (6) BC-dried biomass. Gel B: (1) SB-wet biomass; (2) SB-dried biomass; (3) SBP-wet biomass; (4) SBP-dried biomass; (5) SBV-wet biomass; and (6) SBV-dried biomass. Electrophoresis was carried out using Mini-protean TGX 4–20% precast gels (Bio-Rad Laboratories). Protein bands were stained using Coomassie brilliant blue G-250. Each well was loaded with 20 μg of protein. B, 5% presalting brine at native pH (6.5); BP, 5% presalting brine acidified to pH 4.7; BC, 5% presalting brine acidified to pH 4.7 and flocculated using carrageenan; SB, spice brine at native pH (5.8); SBP, spice brine acidified using 1 N HCl to pH 4.2; and SBV, spice brine acidified using vinegar brine to pH 4.5 followed by addition of 1 N HCl to pH 4.2.

The polypeptide profiles of biomasses obtained from 5% presalting brine ranged between 13 and 215 kDa (Figure 2A). In the lower molecular weight (LMW) range (≤50 kDa), bands were found at 13, 20, 26, 31, 35, 37, 41, and 50 kDa, whereas in the higher molecular weight (HMW) range (>50 kDa), bands were found at 55, 73, 94, 151, and 215 kDa, the latter tentatively identified as the myosin heavy chain. The most intense bands in all biomasses from 5% PSB were found at 35 and 41 kDa (Figure 2A). The bands at 41 and 50 kDa were tentatively identified as actin and desmin, respectively, which agree with the previous observations^33^ on presalting brines. The band at 20 kDa, with high intensities for both B and BP, was tentatively identified as the myosin light chain. Interestingly, the intensity of both heavy and light myosin bands was lower in BC compared to that in B and BP. Biomasses from spice brine, contrary to biomasses from 5% salt brine, possess visible bands only below 50 kDa, at 13, 25, 26, 35, 37, 41, and 48 kDa. This is in agreement with earlier reported polypeptide profiles of brine from spice-salted herring having a molecular weight span of 20–48 kDa (Stefánsson, Nielsen, and Gudmundsdottir).^2b^ Bands at 13, 35, 37, and 41 kDa were present in biomasses from both 5% presalting brine and spice brine (Figure 2B). The intensity of the bands at 25, 26, 35, and 37 kDa was enhanced when the pH of the spice brine was adjusted prior to DAF, while bands at 41 and 48 kDa decreased. The former indicates that these polypeptides precipitated at low pH, providing higher recovery during DAF. The absence of polypeptides above 50 kDa in the spice brine compared to that of 5% presalting brine was most likely due to proteolysis occurring during the prolonged marination of herring in spice brine.

### 3.4 Oscillatory Dynamic Properties of Freeze-Dried Recovered Biomasses

**Figure 3:**
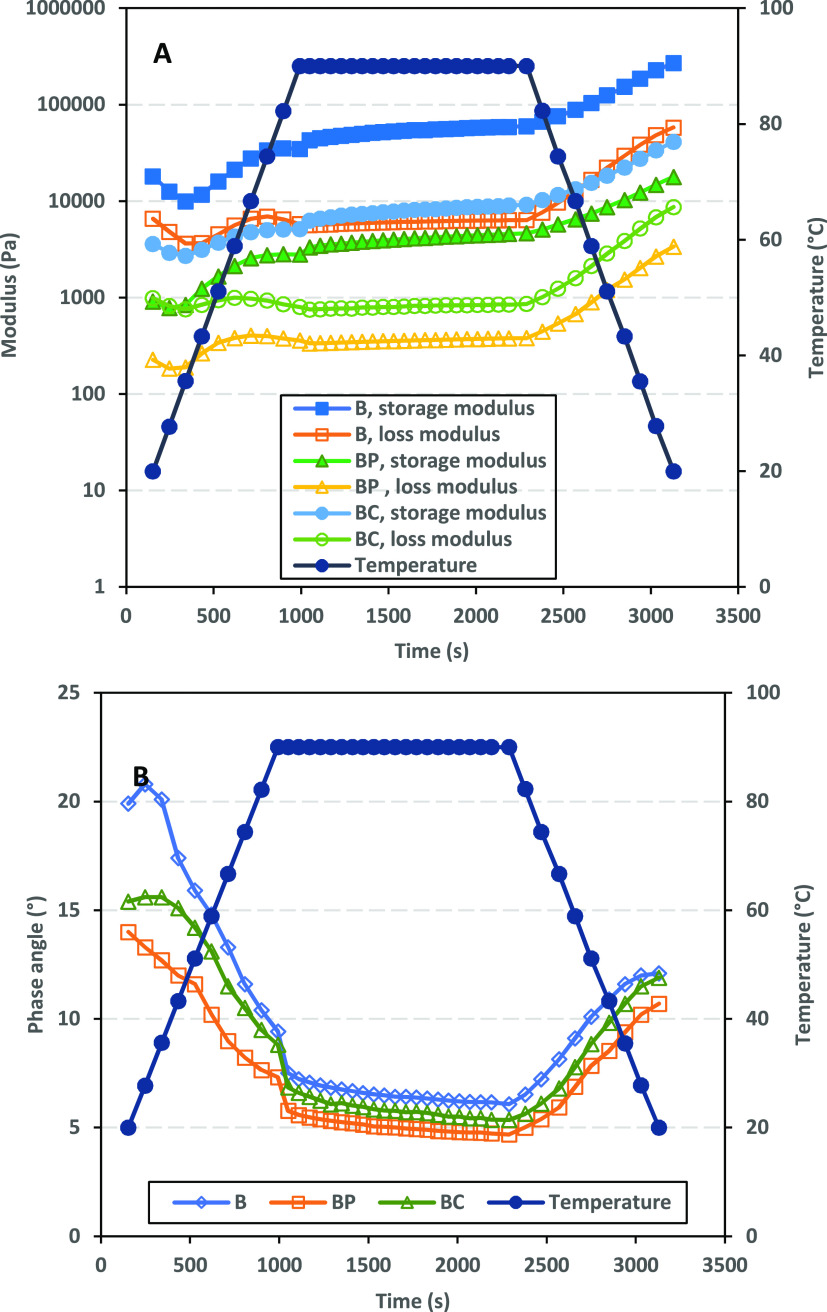
Storage (*G*′) and loss (*G*″) modulus (A) and phase angle (B) of freeze-dried biomasses recovered upon treatment of 5% presalting brine with the F-DAF technique. B, 5% presalting brine at native pH (6.5); BP, 5% presalting brine acidified to pH 4.7; BC, 5% presalting brine acidified to pH 4.7 and flocculated using carrageenan.

Dynamic rheology has been extensively used to study heat-induced gelation of fish myofibrillar proteins. The elastic or storage modulus (*G*′) shows development of an elastic gel network, as it measures the energy recovered per cycle of sinusoidal shear deformation. Hence, by monitoring changes in *G*′, it is possible to monitor protein thermal gelation. The dynamic viscoelastic results from gels made with B, BP, and BC are presented in Figure 3A. The *G*′ index initially decreased from 20 to 35 °C, after which it increased gradually for all samples. The increase which was initiated at 35 °C could be attributed to denaturation of the myosin head. The *G*′ index increase for gels made from BP and BC biomass continued to 66 °C; however, in the gel from B biomass, the increase progressed up to 74 °C. The low *G*′-temperature for BP and BC could be due to protein conformational changes occurring during the acidification and flocculation steps. Multidomain myosin rods produce an extended 3D network when heated to 65 °C and thus participate in the development of a gel network. For instance, when subjecting the herring muscle to heating, four major peaks between 39 and 74 °C were associated with the denaturation of the myofibrillar, myosin, sarcoplasmic protein, and connective tissues.^34^ The next minor increase in *G*′ was identified at 90 °C, which then remained stable with heating at 90 °C, after which it increased again during cooling to 20 °C. Gels made with B, BP, and BC showed similar *G*′ patterns, whereas the *G*′ of the gel from the B biomass was always one order of magnitude higher in comparison to the *G*′ of BP and BC biomass-derived gels. This could be due to the differences in polypeptides of B compared to BP and BC biomasses (Figure 2), for instance, the higher intensity of myosin and the presence of bands at 31 and 35 kDa. Also, the lower ash and higher lipid contents of B compared to those of BP and BC could play a role. The *G*′ index of BC-derived gels was intermediate compared to that of gels made of B and BP. The *G*′ pattern in our study varied greatly from that reported by Abdollahi et al. when subjecting paste made from silver carp and kilka protein isolates and surimi to thermal treatment,^24^ something which could be due to different protein profiles, more salt, and higher/lower lipid levels in our biomasses.

The elastic modulus (*G*′) was higher than the loss modulus (*G*″), which indicates that the ability of the pastes to form elastic gels was higher than their viscous characteristics (Figure 3A). The phase angle (δ) values, indicating the proportion of viscosity to elasticity of the gels, are presented in Figure 3B. A phase angle of 90° corresponds to a viscous response, while δ = 0 indicates an elastic response. When 0 < δ < 90°, the material can be considered viscoelastic. The decline in the phase angle started at 20 °C for gels made with BP, while that of BC and B started at 35 °C, indicating the viscous nature of the gels. In BP samples, the decline was observed to start already at 20 °C, which indicates that the formation of an elastic material has occurred already at a relatively low temperature. In other words, protein recovery initiated by acidification promoted protein aggregation and allowed the formation of an elastic gel at lower temperature. The initial phase angle of B, BP, and BC started within a wide range, at 19.9°, 15.4°, and 14°, respectively, whereas heating at 90 °C caused the initial phase to end up in a narrow range, 6.0°–4.6°. This indicated that despite the significant variation in the *G*′ and *G*″ of the three gels, they were very similar in relation to viscoelastic properties.

### 3.5 Sensory Evaluation of Freeze-Dried Biomasses

**Figure 4:**
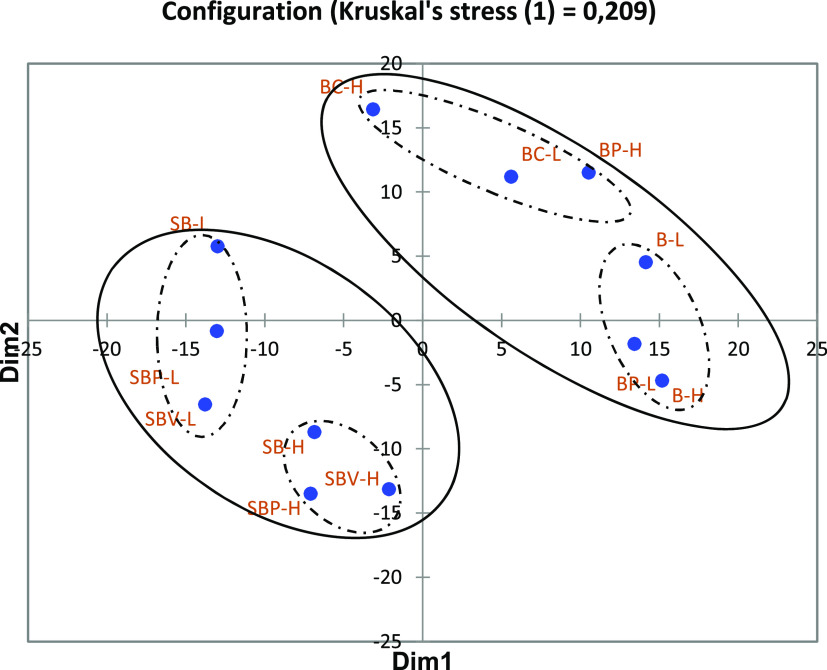
MDS plot illustrating the distribution of freeze-dried biomasses based on the similarities in the sensory profile using the sorting task. The circles represent the clusters obtained from HCA for the sensory evaluation. B, 5% presalting brine at native pH (6.5); BP, 5% presalting brine acidified to pH 4.7; BC, 5% presalting brine acidified to pH 4.7 and flocculated using carrageenan; SB, spice brine at native pH (5.8); SBP, spice brine acidified using 1 N HCl to pH 4.2; and SBV, spice brine acidified using vinegar brine to pH 4.5 followed by addition of 1 N HCl to pH 4.2.

Freeze-dried biomasses recovered from 5% presalting brine and spice brine upon F-DAF were suspended in water at 3 and 5% (w/v) representing low (L) and high (H) concentrations of proteins, respectively. Samples (B-L, B-H, BP-L, BP-H, BC-L, BH, SB-L, SB-H, SBP-L, SBP-H, SBV-L, and SBV-H) were profiled from a sensory point of view by 13 trained panelists using a free sorting task. Samples were grouped based on their similarities with respect to smell and taste, and groups were described using a list of attributes. Figure 4 displays the results of the sensory perception of the samples. Agreement among panelists was assessed by the stress function. The more the stress value converges to 0, the higher the correspondence. Even though Kruskal^35^ stated that only a stress value lower than 0.1 can be considered for evaluation, a stress value of 0.2 is currently acceptable in food sensory.^36^ The stress value for the discrimination space was 0.209, which is slightly over the limit but still acceptable, meaning that panelists perceived similarities and dissimilarities in a similar way. Samples were significantly (*p* < 0.05) perceived differently depending on the source of the powder (5% presalting brine or spice brine), suggesting that the sensory characteristics of the extracted powders are dependent on the composition of the initial brine in which herring was presalted or marinated. When the powders originated from the spice brine, the H-samples (i.e., 5%) were perceived differently from L-samples at 3%. This was however not the case for the powders originating from the 5% presalting brine.

**Figure 5:**
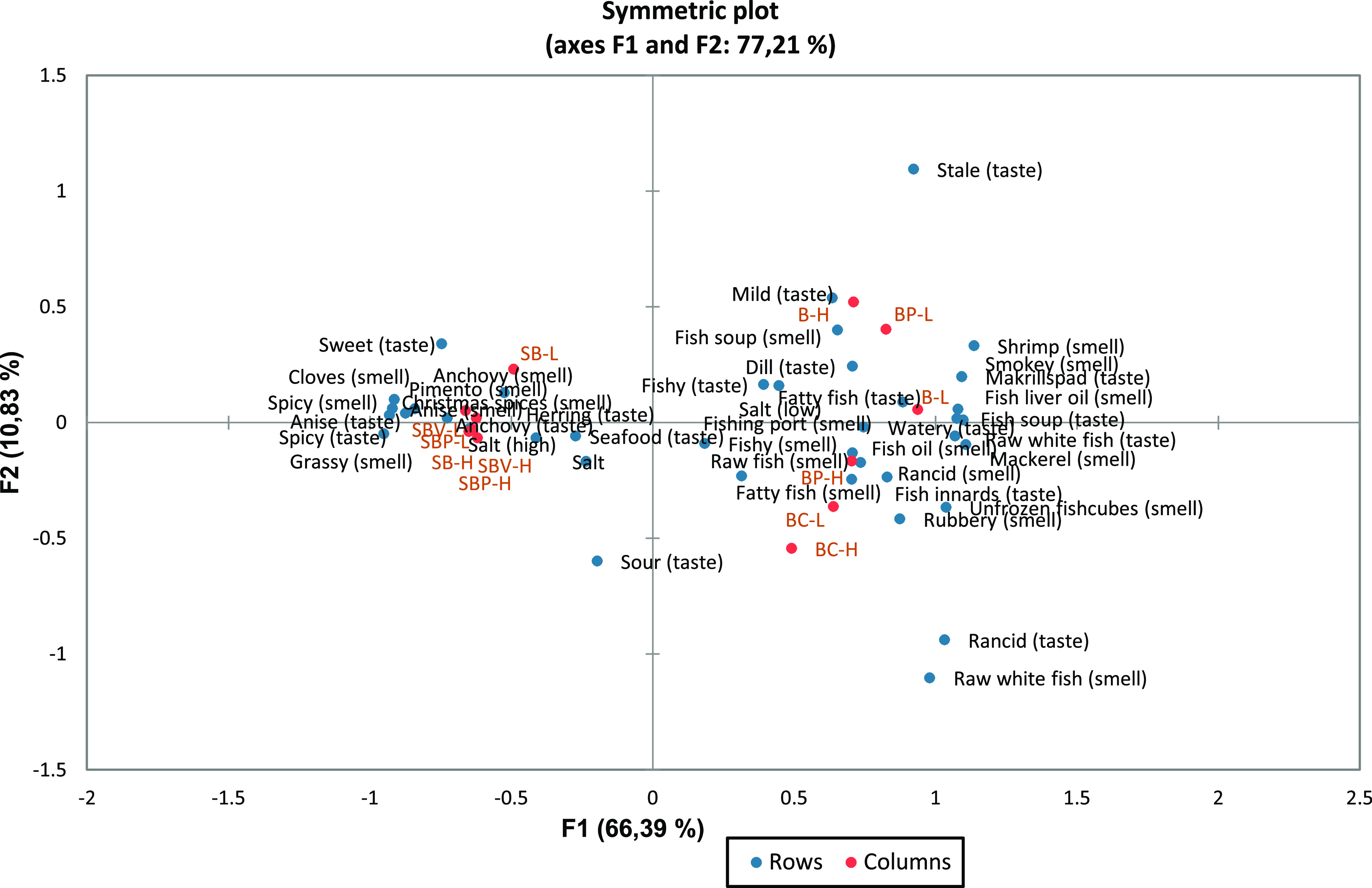
PCA plot illustrating how the freeze-dried biomasses were distributed based on the attributes of powders dissolved in water using the sorting task. B, 5% presalting brine at native pH (6.5); BP, 5% presalting brine acidified to pH 4.7; BC, 5% presalting brine acidified to pH 4.7 and flocculated using carrageenan; SB, spice brine at native pH (5.8); SBP, spice brine acidified using 1 N HCl to pH 4.2; and SBV, spice brine acidified using vinegar brine to pH 4.5 followed by addition of 1 N HCl to pH 4.2.

The sorting task is easily applicable to obtain a quick overview of different products and their similarities/dissimilarities. In general, the suspended powders were found to be quite similar, within each type of brine, particularly for the spice brine samples. The spice brine samples (SB, SBP, and SBV) were mostly characterized by anchovy- and spice-related attributes, whereas the 5% presalting brine-derived samples (B, BP, and BC) were characterized by fish and seafood attributes (Figure 5). Regarding the latter two, lipid oxidation could play an important role in their formation. The spice brine samples were characterized by characteristics such as Christmas-like spices, anise, or pimiento, and the perception of saltiness played an important role when grouping the samples. The sensory notes in 5% presalting brine-derived samples are distinguishable between them with BP-H, BC-L, and BC-H presenting more frequently rancid, raw, and more intense notes, while the rest of the samples had milder, smokey, and more cooked notes (“soup”). Thus, due to the distinctive differences in their sensory characteristics, biomasses from 5% presalting brine and spice brine could be incorporated in different food items to enhance seafood aromas or those derived from the spices.

## 4 Conclusions

Our study demonstrated that the F-DAF technique with carrageenan-based flocculation was highly promising to recover a protein-enriched biomass from 5% presalting brine; the protein yield was as high as 78%. This was a significant improvement compared to acid-induced precipitation alone plus DAF (20% yield) or just DAF. With spice brine, carrageenan did not function as a flocculant due to the high salt content, and protein yields were low both with and without acidification prior to the DAF (2–12%). A prolonged period of DAF or the use of a flotation unit with higher capacity could be tested in future studies to potentially increase the protein yield with spice brine. Freeze-dried biomasses recovered from 5% presalting brine using acidification, carrageenan, and DAF contained 43% protein, 20% lipids, and 20% ash, the latter reflecting a high salt content. In addition, the EAA content was 45% of the total amino acids and the LC n-3 PUFA and LC MUFA contents were 20% and 43% of the total fatty acids, respectively. Dried biomass from spice brine recovered with vinegar brine, HCl, and DAF contained 16% protein, 9% lipid, and 45% ash, the latter reflecting an even higher salt content. The biomass however contained proteins with nutritionally adequate levels of EAA (45% in total) together with fatty acids having 14% LC n-3 PUFA and X% LC MUFA. Polypeptide profiles reflected, for example, that spice brines were recovered after long-term proteolytic ripening of herring and thus only contained polypeptides <50 kDa. Oscillatory dynamic properties revealed that the gel from BC was more elastic than the gels derived from B and BP. Based on the sensory evaluation, spice brine-derived biomasses were characterized by anchovy- and spice-related attributes, while biomasses from 5% presalting brine were characterized by fish and seafood attributes. Additionally, for their potential as nutritious protein ingredients, the high saltiness in all biomasses makes them interesting as food-flavoring agents.

Viewed against the many advantages of the DAF technique, e.g., it is cheap, effective, robust, and often already installed in food-processing companies for in-house precleaning of wastewater, our study reveals that combining this technique with food-grade flocculation is a promising route to extract values from the currently wasted seafood side streams. A main challenge is however to find less costly food-grade flocculants. With the currently used high-quality carrageenan, which was not bought in bulk quantities, the price to treat 1 m^3^ of presalting brine would be 50 euro, which would be too high for a low-value raw material. It is thus recommended to further explore different F-DAF setups as a route to minimize nutrient losses from the seafood value chain and to gradually turn the costs currently related to process water discharge into an income.
